# Larvicidal Potential of the Halogenated Sesquiterpene (+)-Obtusol, Isolated from the Alga *Laurencia dendroidea* J. Agardh (*Ceramiales*: *Rhodomelaceae*), against the Dengue Vector Mosquito *Aedes aegypti* (*Linnaeus*) (*Diptera*: *Culicidae*)

**DOI:** 10.3390/md14020020

**Published:** 2016-01-25

**Authors:** Orlando Salvador-Neto, Simone Azevedo Gomes, Angélica Ribeiro Soares, Fernanda Lacerda da Silva Machado, Richard Ian Samuels, Rodrigo Nunes da Fonseca, Jackson Souza-Menezes, Jorge Luiz da Cunha Moraes, Eldo Campos, Flávia Borges Mury, José Roberto Silva

**Affiliations:** 1 Laboratório Integrado de Bioquímica Hatisaburo Masuda (LIBHM); Núcleo em Pesquisas Ecológicas e Desenvolvimento Sócio-Ambiental de Macaé (NUPEM), Universidade Federal do Rio de Janeiro, Macaé, Rio de Janeiro 27965-045, Brazil; orlando_neto.88@hotmail.com (O.S.-N.); simoneazgomes@yahoo.com.br (S.A.G.); rodrigo.nunes.da.fonseca@gmail.com (R.N.F.); jacksonmenezes@gmail.com (J.S.-M.); jorgemoraes@bioqmed.ufrj.br (J.L.C.M.); eldocampos@yahoo.com.br (E.C.); flaviamury@gmail.com (F.B.M.); 2 Grupo de Produtos Naturais de Organismos Aquáticos (GPNOA), Núcleo em Ecologia e Desenvolvimento Sócio-Ambiental de Macaé (NUPEM), Universidade Federal do Rio de Janeiro, Macaé, Rio de Janeiro 27965-045, Brazil; angelica.r.soares@gmail.com (A.R.S.); fernandanupem@yahoo.com.br (F.L.S.M.); 3 Laboratório Integrado de Ciências Morfofuncionais (LICMF), Núcleo em Ecologia e Desenvolvimento Sócio-Ambiental de Macaé (NUPEM), Universidade Federal do Rio de Janeiro, Macaé, Rio de Janeiro 27965-045, Brazil; 4 Laboratório de Entomologia e Fitopatologia, Universidade Estadual do Norte Fluminense Darcy Ribeiro, Campos dos Goytacazes, Rio de Janeiro 28013-602, Brazil; richardiansamuels@gmail.com; 5 Instituto Nacional de Ciência e Tecnologia em Entomologia Molecular, (INCT-EM), Rio de Janeiro 21941-590, Brazil

**Keywords:** *Laurencia dendroidea*, *Aedes aegypti*, larvicide, sesquiterpenes, (+)-obtusol, (−)-elatol, oxidative stress

## Abstract

Dengue is considered a serious public health problem in many tropical regions of the world including Brazil. At the moment, there is no viable alternative to reduce dengue infections other than controlling the insect vector, *Aedes aegypti* Linnaeus. In the continuing search for new sources of chemicals targeted at vector control, natural products are a promising alternative to synthetic pesticides. In our work, we investigated the toxicity of a bioactive compound extracted from the red alga *Laurencia dendroidea* J. Agardh. The initial results demonstrated that crude extracts, at a concentration of 5 ppm, caused pronounced mortality of second instar *A. aegypti* larvae. Two molecules, identified as (−)-elatol and (+)-obtusol were subsequently isolated from crude extract and further evaluated. Assays with (−)-elatol showed moderate larvicidal activity, whereas (+)-obtusol presented higher toxic activity than (−)-elatol, with a LC_50_ value of 3.5 ppm. Histological analysis of the larvae exposed to (+)-obtusol revealed damage to the intestinal epithelium. Moreover, (+)-obtusol-treated larvae incubated with 2 µM CM-H_2_DCFDA showed the presence of reactive oxygen species, leading us to suggest that epithelial damage might be related to redox imbalance. These results demonstrate the potential of (+)-obtusol as a larvicide for use against *A. aegypti* and the possible mode of action of this compound.

## 1. Introduction

The mosquito *Aedes aegypti* is an important vector of dengue and yellow fever [1,2]. It has also been implicated in the transmission of Chikungunya and Zika virus [3,4]. Dengue is one of the most important arthropod-born viral diseases and a major public health concern. The World Health Organization estimates that there are around 100 million cases of dengue diagnosed annually worldwide [2]. Currently, there are no vaccines against dengue; therefore, the only strategy available to reduce the incidence of the disease is the control of the insect vector. Current control methods rely on the application of chemical insecticides, which has been the basis of reducing the frequency of dengue epidemics over many decades, however with varied success rates. There are four main classes of insecticides which are widely used: organochlorines, carbamates, organophosphates and pyrethroids. The excessive use of chemical control methods has led to the selection of physiological, behavioral, and biochemical resistance mechanisms [5]. As an alternative to chemical control, the use of natural enemies in biological control programs has proven to be efficient in the case of spore-forming bacteria such as *Bacillus thuringiensis* (*Bt*) Berliner [6]. Entomopathogenic fungi are also promising control agents [7,8,9], as are invertebrates [10,11,12] and fish [13].

Even though natural enemies are very important biological control agents, reducing environmental contamination when compared to the use of chemical insecticides, many present limitations such as high cost and instability that leads to low persistence in the field [14]. However, there are new approaches that can be applied to increase the efficiency of mosquito control. For example, combining entomopathogenic fungi with vegetable and synthetic oils has been shown to increase the persistence of the fungus *Metarhizium anisopliae* (Metchnikoff) sorokin under field conditions when tested against adult *A. aegypti* [15].

The search for natural products with potential for use in vector control has gained increased attention. Plants are well known to produce a wide range of compounds with activity against phytophagous insects and plant pathogens. Pyrethroids, for example, are an important class of synthetic insecticides developed from pyrethrum, originally isolated from Chrysanthemum flowers. Many authors have shown the efficiency of plant extracts and essential oils against larval stages of mosquitoes [16,17,18,19]. The use of plants as a source of vector control compounds is now well accepted since these are usually eco-friendly molecules with no negative effects on the environment. Plant derived bioactive compounds are structurally diverse with novel modes of action and many are currently being screened for insecticidal activity in the search for new larvicidal compounds. Compounds such as neem can also be used in integrated vector management, as they had no negative effect on entomopathogenic fungi and when used at very low concentrations, increased the efficiency of the fungus when tested against *A. aegypti* [20].

Seaweeds are known to be rich sources of important bioactive compounds with a range of effects such as anti-cancer [21,22,23], anti-parasitic [24,25,26,27] and antibacterial properties [28,29,30]. In addition, seaweed extracts also have insecticidal activity [31,32,33,34]. Because of their effectiveness against mosquitoes and lack of deleterious effects on the environment, seaweed bioactive compounds are promising models for new synthetic insecticides. Previous studies reported that seaweed-derived compounds displayed insecticidal activity, especially against larval stages of *Culex pipiens pallens* Coquillett [35,36,37,38,39]. Recently, extracts of seaweeds from the northwest coast of Brazil were shown to present larvicidal activity against *A. aegypti* [40]. The halogenated sesquiterpene (−)-elatol was shown to be respsonsbile for this insecticidal activity.

Red algae of the genus *Laurencia* J. V. Lamouroux (*Rhodomelaceae*) occur in temperate to tropical coastal regions of the world, inhabiting intertidal and subtidal areas. These algae are rich sources of secondary metabolites with high structural diversity, mainly halogenated terpenes and C15-acetogenins [21,41,42]. Apart from their potential as sources of new drugs, some compounds also play an important role in chemical defenses against herbivores, and fouling organisms [43,44]. Here we report the larvicidal activity of a crude algal extract and the biological activity of two halogenated sesquiterpenes isolated from *L. dendroidea*. In addition, the physiological and morphological effects of (+)-obtusol, the most toxic of the two compound against *A. aegypti* larvae, were also investigated. The results demonstrated that the midgut is an important site of action of (+)-obtusol. Seaweed-derived compounds such as (+)-obtusol are potential models for the design of eco-friendly insecticides. Understanding the mode of action of (+)-obtusol is important when considering its putative use in combination with another insecticides acting at different sites with the potential for synergistic effects. The combined use of traditional insecticides and plant bioactive compounds may hamper the development of pesticide resistance and reduce the concentrations needed for effective control.

## 2. Results and Discussion

Research on seaweed-derived molecules has recently gained increased interest since they represent a source of diverse biologically active compounds. Importantly, insecticidal activities have been described from seaweed extracts [31,32,33,34,45]. Here we describe the potential larvicidal activity of extracts derived from *L. dendroidea*.

**Figure 1:**
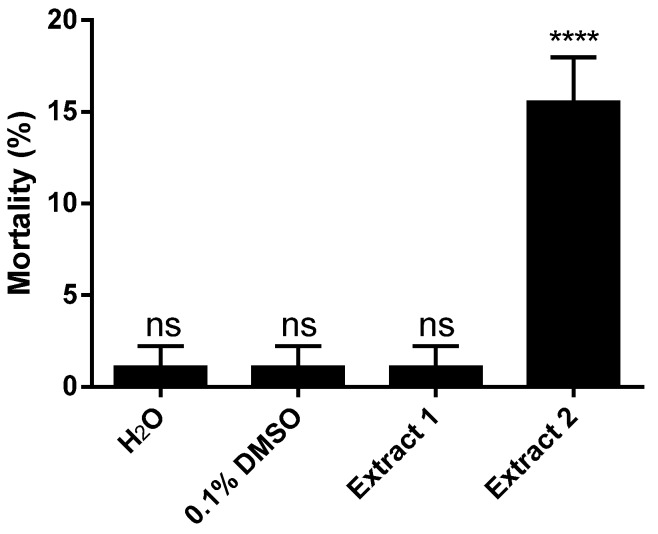
Larvicidal activity of crude extracts from *Laurencia. dendroidea* collected from two different localities against *Aedes aegypti*, Rockefeller strain. Extract 1 was derived from seaweed collected at “Azeda” beach in Búzios and the Extract 2 was derived from seaweed collected at “Vermelha” beach in Parati. Five parts per million of each extract were used in the experiments. Results are means of three independent experiments (ANOVA, followed by Tukey’s multiple comparisons test; **** *p* < 0.0001; ns, denotes no significant difference).

Initially, two crude extracts were screened. The samples were collected from two different locations along the Rio de Janeiro coastline, namely Azeda beach in Búzios and Vermelha beach in Parati. The Vermelha beach crude extract presented significant larvicidal activity, as shown in Figure 1. However, the extract of *L. dendroidea* collected at Azeda beach had very low larvicidal activity and therefore was not used in further studies here.

**Figure 2:**
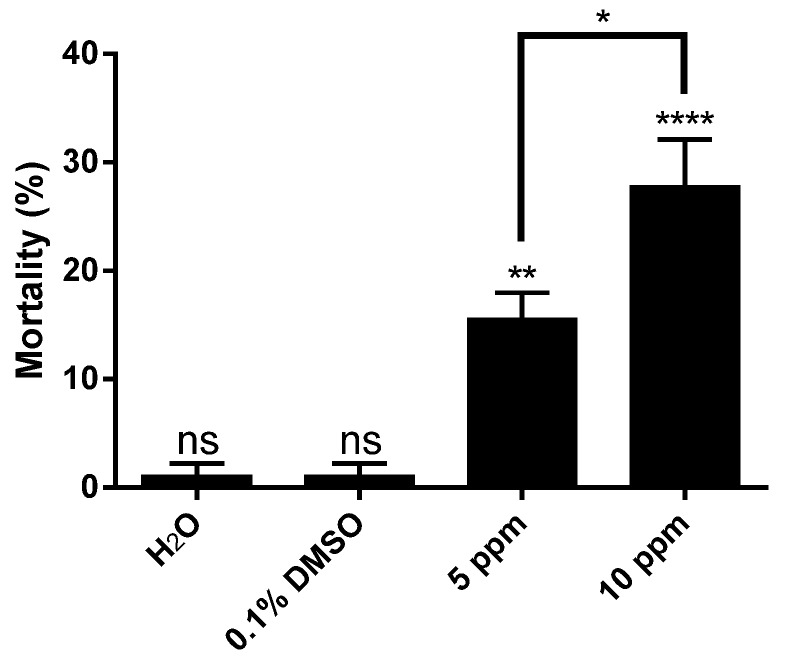
Larvicidal activity of different concentrations of *Laurencia dendroidea* crude extract collected at Vermelha beach in Parati and tested against *Aedes aegypti*. The results are means of three independent experiments (ANOVA, followed by Tukey’s multiple comparisons test; * *p* ≤ 0.012; ** *p* ≤ 0.003; **** *p* < 0.0001; ns, denotes no significant difference).

The distinct larvicidal activities observed suggest the existence of qualitative and/or quantitative differences in metabolite content between locations [46,47,48]. Further GC-MS analysis revealed that both extracts displayed the same major compounds. However, the remaining chromatograms were not absolutely correlated (Figure S1). Therefore, the synergistic/additive effects of different compounds in the crude extracts could also have influenced larvicidal activity in samples from Vermelha beach. The larvicidal effect of Vermelha beach extracts were shown to act in a dose-dependent manner (Figure 2).

Previous studies also evaluated the larvicidal effect of seaweed extracts [33]. The results showed that extracts of *Cheatomorpha antenina* Bory and *Sargassum wightii* Greville were effective against a wild population of *A. aegypti.* However, higher LC_50_ values were determined for the different extracts, in comparison with other seaweed extracts previously reported, ranging from 415 to 516 ppm [33]. The LC_50_ values showed that the toxicity of the *C. antenina* extract varied depending on the solvent used in the extraction: when using benzene, the LC_50_ was 448 ppm, 482 ppm when using acetone and 516 ppm when using methanol. For *S. wightii*, the LC_50_ was 415 ppm when using methanol, 448 ppm with acetone and 473 ppm with benzene [33]. However, the authors did not mention which larval stage was used in their assays.

The screening of a range of seaweed species (*Ulva lactuca* Linnaeus, *Caulerpa racemosa* (Forsskål) J. Agardh, *Sargassum microcystum* J. Agardh, *Caulerpa scalpelliformis* (R. Brown ex Turner) C. Agardh, *Gracilaria corticata* (J. Agardh) J. Agardh, *Turbinaria decurrens* Bory, *Turbinaria conoides* (J. Agardh) Kützing and *Caulerpa toxifolia* (M. Vahl) C. Agardh), showed promising results for *C. racemosa*, with methanolic extracts demonstrating high toxic activity against 4th instar *A. aegypti*, *Culex quinquefasciatus* Say and *Anopheles stephensi* Liston larvae. The LC_50_ values were 0.0556 ppm, 0.0675 ppm and 0.0661 ppm, respectively [34]. A preliminary phytochemical analysis showed that the extracts of these seaweeds presented a variety of constituents such as carbohydrates, saponins, steroids, terpenoids, phenols and proteins [34].

**Figure 3:**
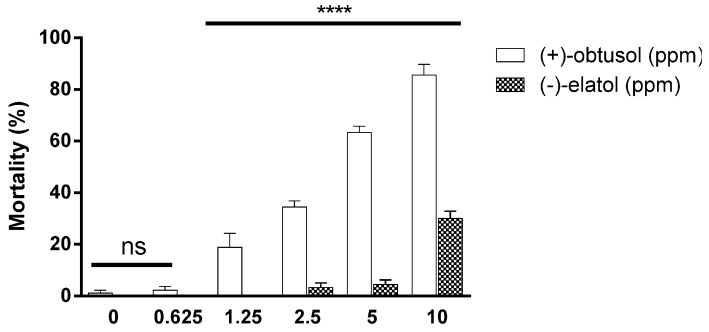
Larvicidal activity of the sesquiterpenes (+)-obtusol and (−)-elatol against *Aedes aegypti* (Rockefeller strain) second instar larvae. Ten larvae were incubated in distilled water in the presence of different concentrations of either obtusol or elatol and mortality was evaluated after 24 h. The control consisted of 0.1% DMSO in water. The results represent mean ± SEM of three independent experiments (ANOVA, followed by Bonferroni’s multiple comparisons test; **** *p* ≤ 0.0001; ns, denotes no significant difference) (see Figure S1).

In order to identify the compounds responsible for the larvicidal activity observed in the present study, the crude extract from *L. dendroidea* collected at Vermelha beach was fractionated, yielding two halogenated sesquiterpenes, identified as (−)-elatol and (+)-obtusol. The toxicity of both pure compounds was evaluated against second instar larvae of *A. aegypti.* (−)-Elatol showed larvicidal activity with 10 ppm killing approximately 30% of the larvae within 24 h (Figure 3). At lower concentrations (−)-elatol was not effective and there was no difference when compared to controls (*p* < 0.0001) (Figure S2). (−)-Elatol is also present in other species of *Laurencia* [27,43,49,50,51,52] and it is believed to play an important ecological role in protecting the seaweed against herbivores and microbial infections [43,44,53,54]. However, the first report of (−)-elatol insecticidal activity was only published recently [40]. The LC_50_ value of 10.7 ppm against *A. aegypti* was similar to that demonstrated here.

**Figure 4:**
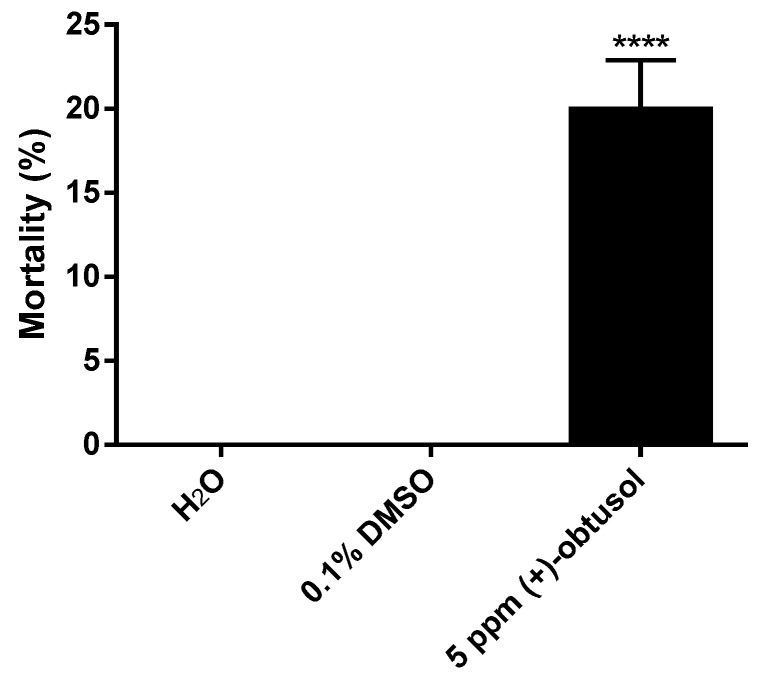
Larvicidal activity of (+)-obtusol against *Aedes aegypti* (Rockefeller strain) forth instar larvae. Ten larvae were incubated in distilled water in the presence of 5 ppm (+)-obtusol and mortality evaluated following 24 h. The controls consisted of water and water containing 0.1% DMSO. The experiment was carried out in three replicas and the results represent mean ± SEM of three independent experiments (ANOVA, followed by Tukey’s multiple comparisons test; **** *p* ≤ 0.0001).

However, bioassays using (+)-obtusol showed that this compound is more toxic to mosquito larvae than (−)-elatol (*p* < 0.0001) (Figure 3). Using a concentration of 10 ppm, (+)-obtusol killed approximately 90% of second instar *A. aegypti* larvae within 24 h (Figure 3). Even at lower concentrations, (+)-obtusol showed a reasonable larvicidal activity (*p* < 0.0001) (Figure S2). It was noted that (+)-obtusol acted in a dose-dependent manner (Figure 3), with a LC_50_ of 3.5 ppm. Even at concentrations as low as 1.25 ppm it still caused approximately 20% larval mortality. The current study showed that (+)-obtusol is present in extracts from Vermelha beach, but absent in the extracts from Azeda beach, accounting for the reduced toxicity of this extract (Figure S1). Additionally, incubation of fourth instar larvae in 5 ppm (+)-obtusol resulted in 20% mortality within 24 h (*p* < 0.0001) (Figure 4). The reduced toxicity of (+)-obtusol (5 ppm) against fourth instar larvae when compared to second instar larvae might be due to the concentration: body mass ratio.

**Figure 5:**
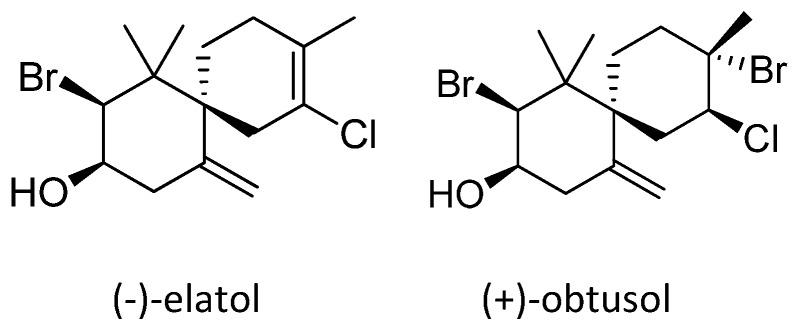
Structures of the isolated sesquiterpenes [27].

Structurally, (−)-elatol and (+)-obtusol (Figure 5) differ only in one double bond and the presence of a bromine [27]. Bianco *et al.* [40] suggested that the presence of a halogen could be very important for larvicidal activity of (−)-elatol. Therefore, the additional bromine in (+)-obtusol could be responsible for the increase in potency when compared to (−)-elatol. However, further studies with a series of structurally similar sesquiterpenes are needed to confirm this hypothesis.

With the aim of excluding the possibility that the observed effects were caused by the vehicle employed here (DMSO), we tested different concentrations of DMSO against second instar larvae. The results showed that DMSO had no lethal effect at the 0.1% concentration used in all the tests here (Figure S3).

**Figure 6:**
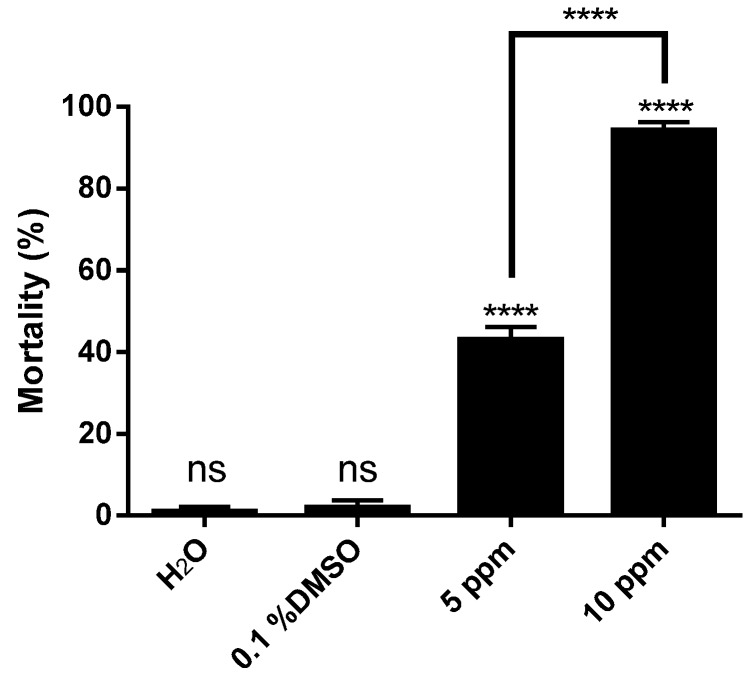
Larvicidal activity of two different concentrations of (+)-obtusol against wild strain *Aedes aegypti*. Ten second instar larvae were incubated in water containing two different concentrations of (+)-obtusol (5 and 10 ppm). The controls consisted of water and water +0.1% DMSO. Mortality was evaluated after 24 h. The results represent mean ± SEM of three independent experiments (ANOVA, followed by Tukey’s multiple comparisons test; **** *p* ≤ 0.0001; ns, no significant difference).

**Figure 7:**
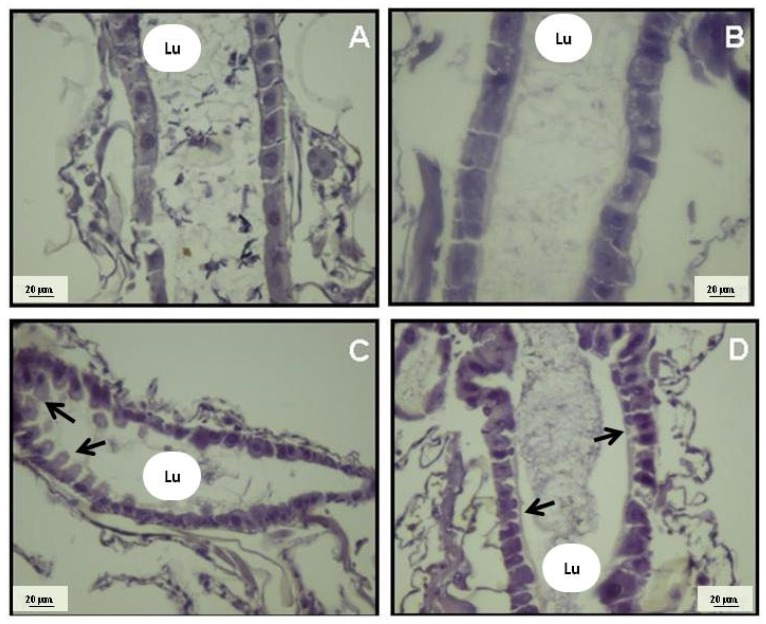
Light micrographs showing the midgut epithelium of second instar *Aedes aegypti* larvae, which had been exposed to 5 ppm (+)-obtusol: (**A**) control larval gut after incubation with water; (**B**) control larval gut after incubation with 0.1% DMSO in distilled water; (**C**) *A. aegypti* (Rockefeller strain) larval gut after incubation with 5 ppm (+)-obtusol in distilled water; and (**D**) wild strain *A. aegypti* larval gut after incubation with 5 ppm (+)-obtusol in distilled water. Lu: Lumen. Arrows point to regions with altered morphology.

In addition to the Rockefeller strain, we tested the susceptibility of a natural population of *A. aegypti* to (+)-obtusol. It was observed that this strain was also susceptible to the effects of (+)-obtusol (Figure 6). Ten ppm of (+)-obtusol killed 95% of the larvae in 24 h, whereas 5 ppm killed approximately 40% of the larvae (*p* < 0.0001). Due to the promising results seen for toxicity of (+)-obtusol against second and fourth instars *A. aegypti* larvae, the physiological and cytotoxic effects of this compound were investigated. The midgut is considered an important site to be analyzed, since it represents the first line of contact with compounds ingested by the larvae, and also where compounds could be concentrated under conditions of continuous exposure. Histological analysis of the larval midgut epithelia showed that (+)-obtusol caused marked changes in cell morphology, both in Rockefeller strain and natural populations of *A. aegypti* (Figure 7C,D, respectively). Cell morphology changed from a rectangular shape observed in the controls, water (Figure 7A) and 0.1% DMSO (Figure 7B), to ovoid with a less organized barrier following exposure to (+)-obtusol (Figure 7C,D). Similar results were reported by Al-Mehmadi and Al-Khalaf [55] when investigating the histological effects of the extract of a Meliaceae plant family, *Melia azedarach* Linnaeus, a known potential source of natural insecticides. According to the techniques used in the present work, it was not possible to correlate the morphological changes to any mitochondria dysfunction. Nor was any vacuolization observed at cellular level. We plan to further investigate these morphological effects.

**Figure 8:**
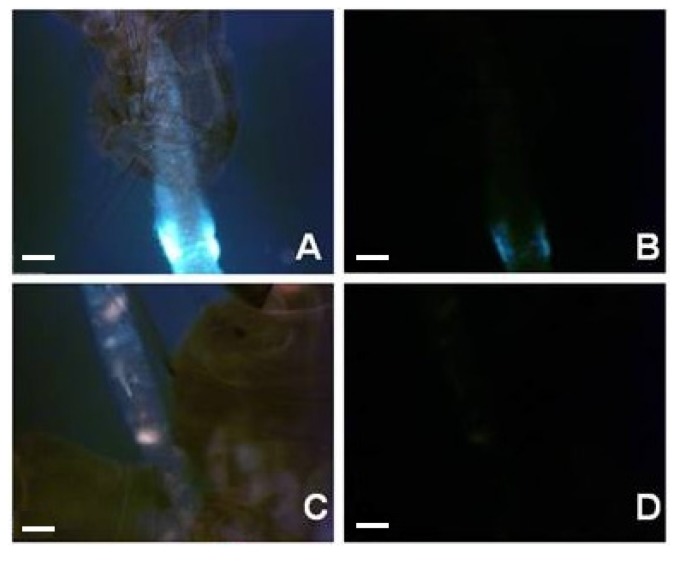

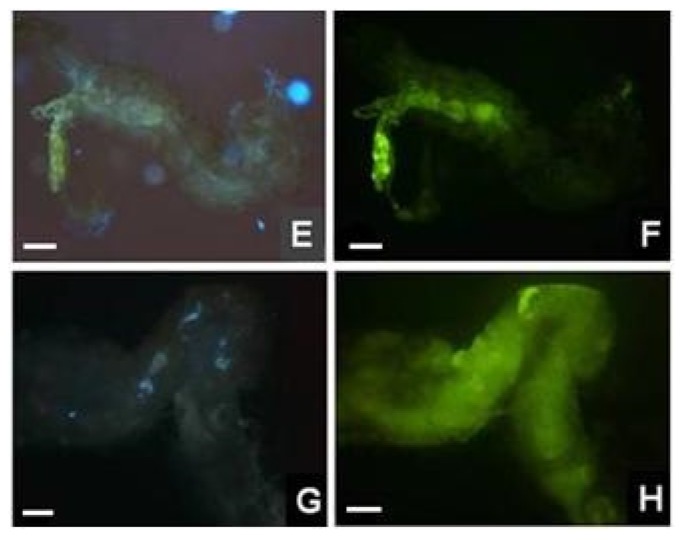
(+)-Obtusol increases ROS level in the larvae. Second instar *Aedes aegypti* larvae (Rockefeller and wild strains), were incubated in water under different conditions; (**A**) bright field image of larvae incubated in distilled water; (**B**) fluorescent control image of larvae incubated in distilled water alone; (**C**) bright field image of larvae incubated in distilled water containing 0.1% DMSO; (**D**) fluorescent control image of larvae incubated in distilled water containing 0.1% DMSO; (**E**) bright field image of second instar larvae (Rockefeller strain), incubated with water containing 5 ppm (+)-obtusol; (**F**) fluorescent image of second instar larvae (Rockefeller strain), incubated with water containing 5 ppm (+)-obtusol; (**G**) bright field image of second instar larva (wild strain), incubated in water containing 5 ppm (+)-obtusol; (**H**) fluorescent image of second instar larva (wild strain), incubated in water containing 5 ppm (+)-obtusol. Scale bars: 100 µm.

**Figure 9:**
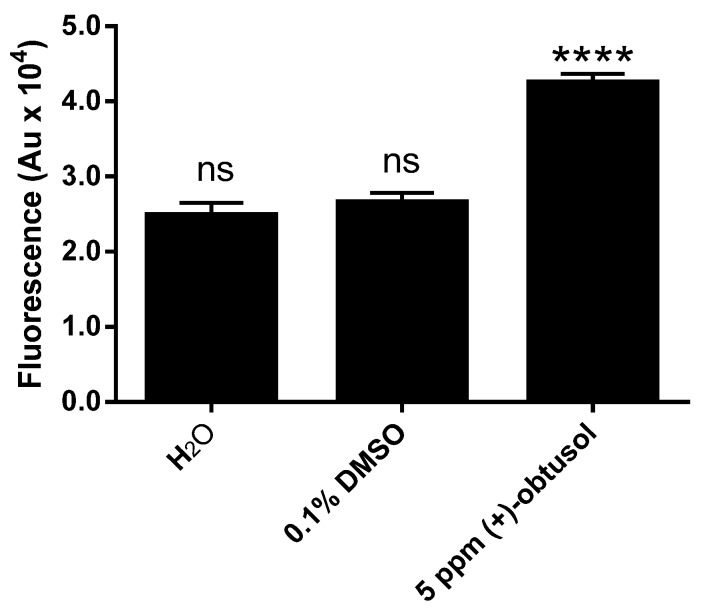
Increase in ROS level in larvae incubated in water in the presence of (+)-obtusol. Second instar larvae of *Aedes aegypti* (Rockefeller strain), previously incubated in water containing 5 ppm (+)-obtusol. The integument was then pricked and larvae incubated in 2 µM CM-H_2_DCFDA in PBS for 20 min. After that time, larvae were homogenized in probe-free PBS and centrifuged at 20,000× *g* for 10 min at 4 °C. The supernatant was analyzed in a Spectrofluorometer (Excitation: 563 nm; Emission: 587 nm). The results represent mean ± SEM of two independent experiments. Each replica consisted of ten larvae (ANOVA, followed by Sidak’s multiple comparisons test; **** *p* ≤ 0.0001).

To investigate the physiological effects following midgut epithelial alterations, an analysis similar to that previously performed by Santos *et al.* [26] was carried out here on *A. aegypti*. Their study showed that (−)-elatol caused vacuolization of *Trypanosoma cruzi* Chagas cytoplasm and alterations in the mitochondrial ultra-structure. In addition, it has been demonstrated that the mitochondrion is a target for (−)-elatol action possibly exacerbating the ROS production [56]. If the mitochondrion is a possible target, then the effects might have been due to redox imbalance. As (+)-obtusol is structurally similar to (−)-elatol, we decided to investigate its effect on the levels of reactive oxygen species (ROS) in *A. aegypti*. Using fluorescence stereo microscopy and an oxidant-sensitive probe (CM-H_2_DCFDA) we observed that 5 ppm (+)-obtusol caused an increase in the levels of ROS in the larvae 24 h after exposure to this compound (Figure 8F,H). Neither 0.1% DMSO (Figure 8D) nor water (Figure 8B) caused any increase in ROS levels. Fluorometric analysis of the larvae exposed to 5 ppm (+)-obtusol confirmed the increased ROS levels (Figure 9).

Our results suggest that the midgut is an important site of action of (+)-obtusol and the increased level of ROS could contribute to larval mortality. This is the first time that morphological and physiological effects have been shown in larvae treated with a seaweed-derived molecule, as previously only physiological effects of compounds derived from terrestrial plants have been described [39,57,58,59]. The data here sheds some light on the site of action of these molecules, which could be investigated as models for design of more efficient vector control agents.

Additionally, the present work corroborated previous reports suggesting that seaweeds are very important sources of insecticides, since they produce bioactive compounds with higher potency than many terrestrial organisms. It is worth mentioning that *L. dendroidea* crude extract from Vermelha beach was more potent in comparison to many of the previously investigated seaweed extracts [39,60,61,62]. It is important to evaluate strategies for utilizing seaweed-derived metabolites to control disease vectors such as *A. aegypti*. One feasible idea is to microencapsulate metabolites with inert materials which could improve delivery. Not least important is the use of a combination of agents with different modes of action. In this case one might use seaweed-derived compound in combination with a *B. thuringiensis* to potentiate their insecticidal activity and reducing the development of pesticide resistance.

## 3. Experimental Section

### 3.1. Maintenance of the *A. aegypti* Colony

*A. aegypti* (Rockefeller strain) were reared in the insectary of the Integrated Biochemistry Laboratory Hatisaburo Masuda. The adult female mosquitoes were kept in plastic cages (30 cm × 20 cm × 20 cm) covered with fine mesh netting at a relative humidity of 70% ± 5% and a photoperiod of 12 h light and 12 h dark. The adults were fed *ad libitum* on 10% sucrose. In order to produce and lay eggs, adult females were fed on mice immobilized in a wire mesh bag and placed in the adult cage. The use of live animal to feed the female mosquitoes was approved by the UFRJ-Macaé Ethical Committee. After feeding on blood, females were allowed to lay their eggs on wet filter paper lining beakers which were half filled with water. For larvae eclosion, mouse food was added to distilled water and allowed to ferment for 24 h in order to reduce oxygen level. After that the, eggs were soaked in this water. Larvae that emerged were kept in distilled water with finely ground mouse chow.

### 3.2. Seaweed Sampling

The red seaweed *L. dendroidea* J. Agardh (*Ceramiales*, *Rhodomelaceae*) was collected from two distinct areas in Rio de Janeiro state: Azeda Beach—Búzios (22°44′33.6″ S, 41°52′55.6″ W) in March 2011 and Vermelha Beach—Parati (23°11′35.0″ S, 44°38′39.0″ W) in April 2011. Botanical identification was carried out by L. M. Gestinari and voucher specimens (Azeda beach: RFA 38846 and Vermelha beach: RFA 36045) were deposited in Rio de Janeiro Federal University Herbarium (RFA).

### 3.3. Extract Preparation and Sesquiterpene Purification

The collected samples were washed in seawater to eliminate associated organisms, air-dried at ambient temperature then milled using a blender and solvent extracted at room temperature. The air-dried algae from each sample, 27 g (Azeda beach) and 111 g (Vermelha beach), were extracted three times with CH_2_Cl_2_ (0.5 L and 2.2 L, respectively) with the assistance of an ultrasonicator. The solvent was removed under reduced pressure, yielding 0.4 g (1.3%) and 2.5 g (2.3%) of dark green oil fractions, respectively.

The Vermelha beach crude extract (1.9 g) was purified by silica gel column chromatography (52.0 g Silicycle Silicaflash F60; 230–400 mesh silica in a glass column: 1.5 cm × 56 cm) using an elution gradient of *n*-hexane-CH_2_Cl_2_, CH_2_Cl_2_-AcOEt and MeOH (200 mL of each mixture). In total, 77 fractions were obtained. Similar fractions were pooled according to TLC patterns into 24 fractions (1–24). Fraction 17 (170 mg) eluted in *n*-hexane:dichloromethane (50:50) and fraction 20 (64 mg) eluted in dichloromethane were identified as (−)-elatol and (+)-obtusol, respectively. (−)-Elatol and (+)-obtusol were identified by comparison of their spectroscopic data with previously reported spectra [27].

(−)-Elatol: colorless oil; [α]_D_ −66.2 (*c* 0.13, CHCl_3_); IR (mineral oil) 3458; 2970; 2947; 1718; 1676; 898; 817; 736 cm^−1^; NMR ^1^H (300 MHz, CDCl_3_): 1.07 (s, H-13); 1.08 (s, H-12); 1.62 (ddd, 13.4; 12.5; 7.6, H-1); 1.81 (ddd, 14.8; 12.5; 2.1, H-2); 1.82 (ddd,13.4; 4.1; 2.1, H-1); 1.96 (ddd, 14.8; 7.6; 4.1, H-2); 2.37 (brd, 16.1, H-5); 2.51 (dd, 14.4; 3.0, H-8); 2.59 (dl, 16.1, H-5); 2.63 (dd, 14.4; 3.0, H-8); 4.14 (ql, 3.0, H-9); 4.60 (d, 3.0, H-10); 4.79 (brs, H-14); 5.12 (brs, H-14).^13^C (75 MHz, CDCl_3_): 19.4 (CH_3_-C15); 20.7 (CH_3_-C13); 24.2 (CH_3_-C12); 25.6 (CH_2_-C1); 29.3 (CH_2_-C2); 38.0 (CH_2_-C8); 38.6 (CH_2_-C5); 43.1 (C_0_-C11); 49.1 (C_0_-C6); 72.2 (CH-C9); 70.8 (CH-C10); 115.9 (CH_2_-C14); 128.0 (C_0_-C3); 124.1 (C_0_-C4); 140.8 (C_0_-C7). EI-MS *m*/*z* (rel int %): 319 (2); 317 (1); 299 (3); 297 (3); 253 (8); 237 (40); 236 (18); 235 (100); 217 (7); 209 (15); 207 (29); 200 (9); 199 (36). See also Figures S7 and S8.

(+)-Obtusol: white amorphous solid; [α]_D_ +9.61 (*c* 0.05, CHCl_3_); IR (KBr) 3465; 2969; 1640; 1441; 907; 813; 792 cm^−1^; NMR ^1^H (300 MHz, CDCl_3_): 1.08 (s, H-13); 1.56 (brs, OH); 1.74 (m, H-1); 1.83 (s, H-15); 1.94 (dd, 14.0; 11.7, H-5); 2.30 (m, H-2); 2.36 (brs, H-5); 2.49 (dd, 14.1; 3.1, H-8); 2.62 (dd, 14.1; 3.1, H-8); 4.10 (sl, H-9); 4.47 (d, 3.0, H-10); 4.70 (dd, 11.7; 2.9, H-4); 5.05 (s, H-14); 5.39 (s, H-14).^13^C (75 MHz, CDCl_3_): 20.6 (CH_3_-C13); 23.9 (CH_3_-C10); 24.2 (CH_3_-C12); 25.6 (CH_2_-C1); 37.1 (CH_2_-C5); 38.5 (CH_2_-C8); 40.5 (CH_2_-C2); 44.2 (C_0_-C11); 50.3 (C_0_-C6); 67.6 (CH-C3); 68.1(CH-C4); 70.1 (CH-C10); 71.9 (CH-C9); 117.8 (CH_2_-C14); 141.2 (C_0_-C7). EI-MS *m*/*z* (rel int %): 319 (25); 318 (17); 317 (100); 316 (13); 315 (76); 299 (17); 297 (18); 235 (23); 217 (12); 200 (18); 199 (47). Also see Figures S4–S6.

### 3.4. Analysis of Larvicidal Activity of Extracts and Pure Compounds

Both crude extracts and purified molecules were solubilized in DMSO (Vetec) giving stock solutions of 10,000 ppm and then stored at −20 °C until use. The assays were conducted according to a protocol adapted from World Health Organization for larvicidal bioassays [63]. Briefly, the experiments were carried out in plastic cups containing 50 mL of distilled water with ten 2nd instar, or ten 4th instar larvae maintained at 28 °C. Different amounts of crude extract or pure molecules were added to the water, as indicated in the figures. Controls consisted of larvae incubated in distilled water only or distilled water containing 0.1% DMSO, the final DMSO concentration in all treatments, taking into account that crude extracts and pure molecules were solubilized in DMSO and then diluted in water. Larvae were observed every 6 h during a 24 h period. Larval mortality was determined when there was no response of larvae to tactile stimulation.

### 3.5. Histological Analysis

For histological analysis, second instar larvae were fixed in 4% paraformaldehyde PBS (pH 7.4) overnight. The samples were then embedded in Paraplast. Five-micrometer sections were obtained using a rotary microtome (Leica RM 2245, Leica, Mannheim, Germany) and stained with hematoxylin and eosin. The sections were analyzed using light microscopy (Nikon 80i, Melville, NY, USA) and images were captured with the aid of a digital camera and appropriate software.

### 3.6. Detection of Reactive Oxidative Species (ROS) in Vivo

In order to detect the presence of ROS *in vivo*, we used a protocol developed by Oliveira *et al.* [64]. Larvae were treated with 5 ppm (+)-obtusol and incubated for 20 min in PBS in the presence of a 2 μM solution of an oxidante-sensitive probe CM-H_2_DCFDA (5-(and-6)-chloromethyl-29,79-dichloro-dihydrofluorescein diacetate, acetyl ester) in PBS at room temperature in the dark. Before incubation larvae were pricked to permit the incubation medium to penetrate the haemocoel. After 20 min, larvae were washed in probe-free PBS and transferred to Petri dishes for epifluorescence stereo microscopy examination using a fluorescent stereo microscope (Leica M205 FA, Leica, Mannheim, Germany) with a green filter (450–490 nm) and fitted with a DFC550 digital camera. The images were acquired with the microscope’s software. After 20 min of incubation, the larvae were homogenized in probe-free PBS and centrifuged at 20,000× *g* for 10 min at 4 °C. The supernatant was analyzed in a Spectrofluorometer (Varian Cary Eclipse, Mulgrave, Victoria, Australia; Filters: Excitation: 563 nm; Emission: 587 nm).

### 3.7. Statistical Analysis

Statistical analyses were carried out using Graphpad Prism Program. One-Way ANOVA with a confidence interval of 99% and Dunett, Tukey, or Sidak’s *post*-*hoc* test were used here. A Two-Way ANOVA was also used, followed by Bonferroni’s *post*-*hoc* test. To calculate LC_50_, we used the Spearman v1.0 program with a confidence interval of 95%.

## 4. Conclusions

In conclusion, the present work described the larvicidal activity of *L. dendroidea* extracts from two coast regions in Brazil (Azeda and Vermelha Beaches). After testing two sesquiterpenes compounds, (−)-elatol and (+)-obtusol, isolated from the active crude extract, we showed that (+)-obtusol is more toxic to *Aedes* larvae than (−)-elatol. We also reported here for the first time the morphological and physiological effects of this seaweed-derived compound on the larval midgut and the results indicated that this is one of the first sites of action of this molecule. From our biochemical analyses, the higher levels of ROS in (+)-obtusol-treated larvae indicate that ROS might be the causative agent of the morphological alternations seen in the midgut tissues. This work demonstrates that seaweeds could represent important sources of new insecticides and (+)-obtusol is a promising candidate as a model for insecticide design.

## Supplementary Files

Supplementary File 1
