# Supplementary material for: Larvicidal Potential of the Halogenated Sesquiterpene (+)-Obtusol, Isolated from the Alga Laurencia dendroidea J. Agardh (Ceramiales: Rhodomelaceae), against the Dengue Vector Mosquito Aedes aegypti (Linnaeus) (Diptera: Culicidae)
Source: Mar Drugs. 2016 Jan 25;14(2):20. doi: 10.3390/md14020020 (PMC4771978; doi:10.3390/md14020020)
Supplement: Supplementary File 1 [file marinedrugs-14-00020-s001.pdf]

Supplementary Materials: Larvicidal Potential of the Halogenated Sesquiterpene (+)-Obtusol, Isolated from the Alga *Laurencia dendroidea* J. Agardh (*Ceramiales*: *Rhodomelaceae*), against the Dengue Vector Mosquito *Aedes aegypti* (*Linnaeus*) (*Diptera*: *Culicidae*)

Orlando Salvador-Neto, Simone Azevedo Gomes, Angélica Ribeiro Soares, Fernanda Lacerda da Silva Machado, Richard Ian Samuels, Rodrigo Nunes da Fonseca, Jackson Souza-Menezes, Jorge Luiz da Cunha Moraes, Eldo Campos, Flávia Borges Mury and José Roberto Silva

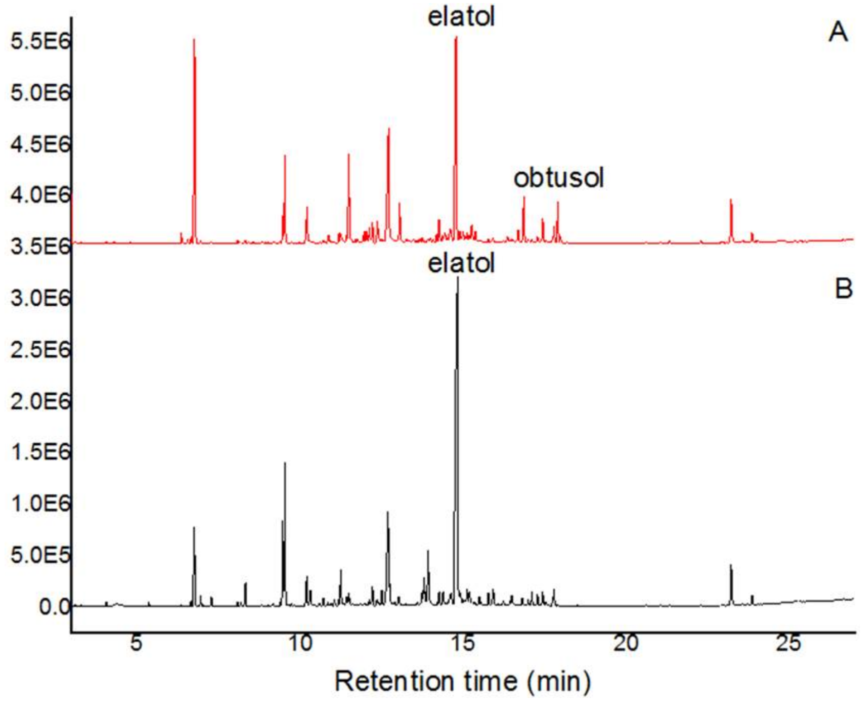

Figure S1. Total ion chromatogram (GC-MS) of CH_2Cl_2 extracts from Vermelha (**A**) and Azeda samples (**B**). Analysis conditions: GCMS-QP2010 Plus Shimadzu in the electron impact mode (70 eV), Rtx-1MS capillary column (60 m × 0.25 mm; film thickness: 0.1 µm, Restek). Temperatures of injector, interface and ion source: 260 °C, 260 °C and 240 °C, respectively. Temperature of the oven was programmed to be maintained at 100 °C for two minutes and then increase at a rate of 8 °C/minute until 300 °C. Helium was used as carrier gas at a flow rate of 1.8 mL/ min. Sample was injected in split mode (1 µL; ratio 1:20). The compounds were identified based on mass spectra and retention time comparison with those of isolated compounds. (+)-Obtusol was not detected in the Azeda beach sample.

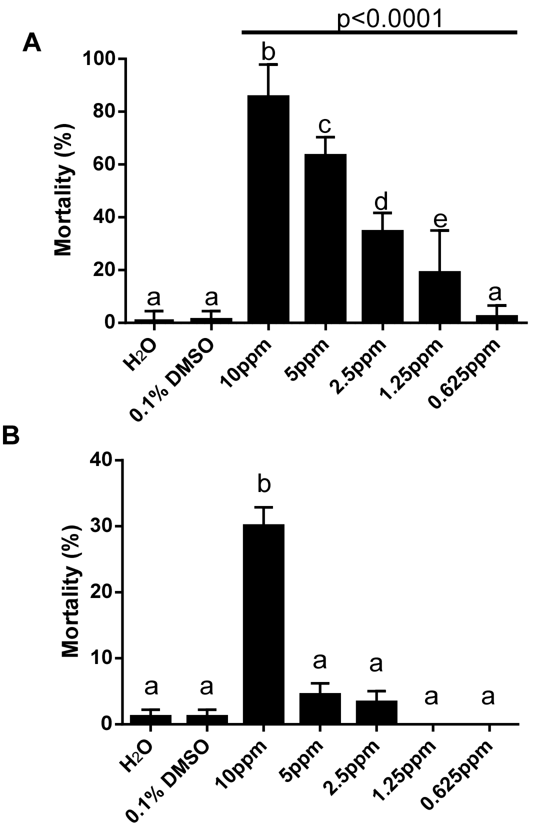

Figure 2. Dose-dependent effect of obtusol and elatol on larval mortality. (**A**) Response to obtusol. All (+)-obtusol concentrations were significantly different from the controls: water or water with 0.1% DMSO (ANOVA, followed by Tukey's multiple comparisons test. $p < 0.0001$), and from one another. Different letters indicated significant differences. The higher the concentration the greater the larvicidal effect, with 10 ppm being significantly more efficient than every other concentration ($p < 0.0001$). At low concentrations, the efficiency was reduced, with 2.5 ppm significantly different from 1.25 ppm ($p = 0.0086$), and 1.25 ppm significantly different from 0.625 ppm ($p = 0.0038$). (**B**) Effect of (−)-elatol on larval mortality. Ten parts per million was significantly different from the controls and also from the other concentrations tested here (ANOVA, followed by Tukey's multiple comparisons test; b, denotes significant difference, $p < 0.0001$; a, denotes no significant difference).

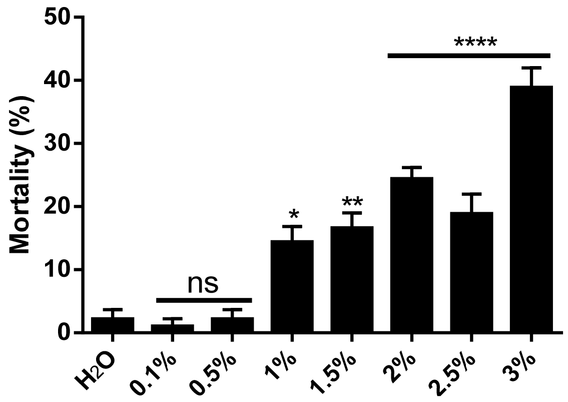

Figure 3. Insecticidal activity of different concentrations of DMSO against second instar *A. aegypti* (Rockefeller strain) larvae. Ten second instar larvae were incubated in distilled water in the presence of different concentrations of DMSO. The control consisted of distilled water alone. The results represent mean ± SEM of three independent experiments. (ANOVA followed by Dunett's multiple comparisons test. * $p = 0.0015$; ** $p = 0.0001$; **** $p < 0.0001$; ns, no significant difference).

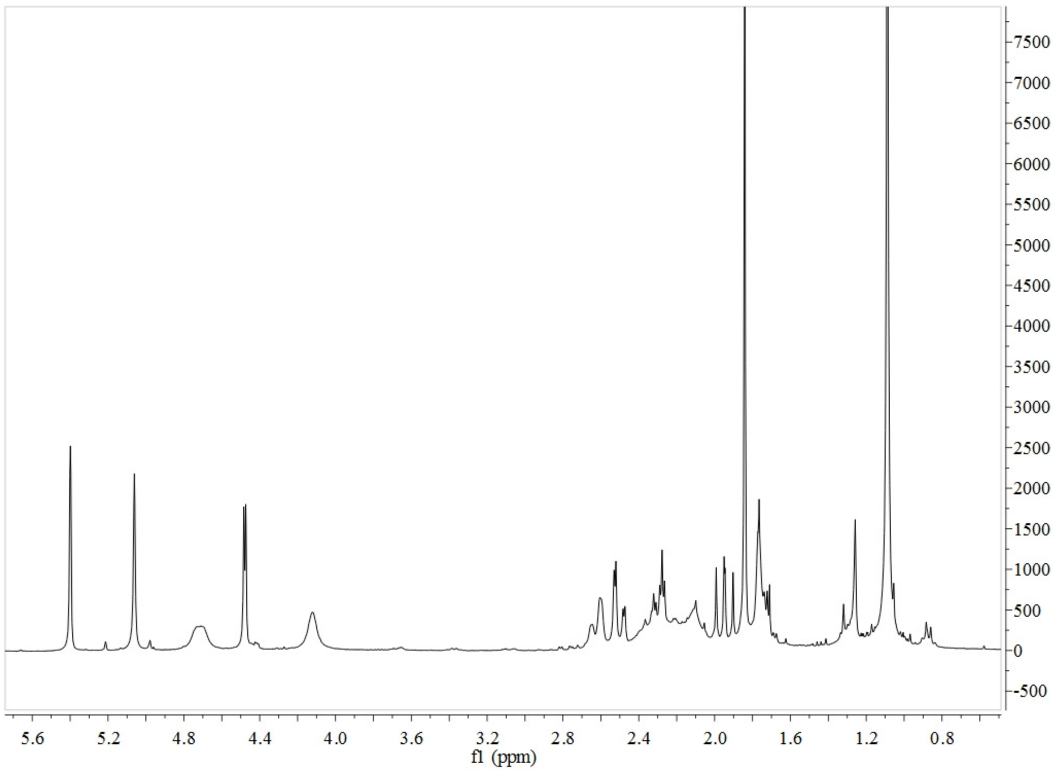

Figure 4. ^{1}H NMR spectra (300 MHz, $CDCl_3$) of (+)-obtusol.

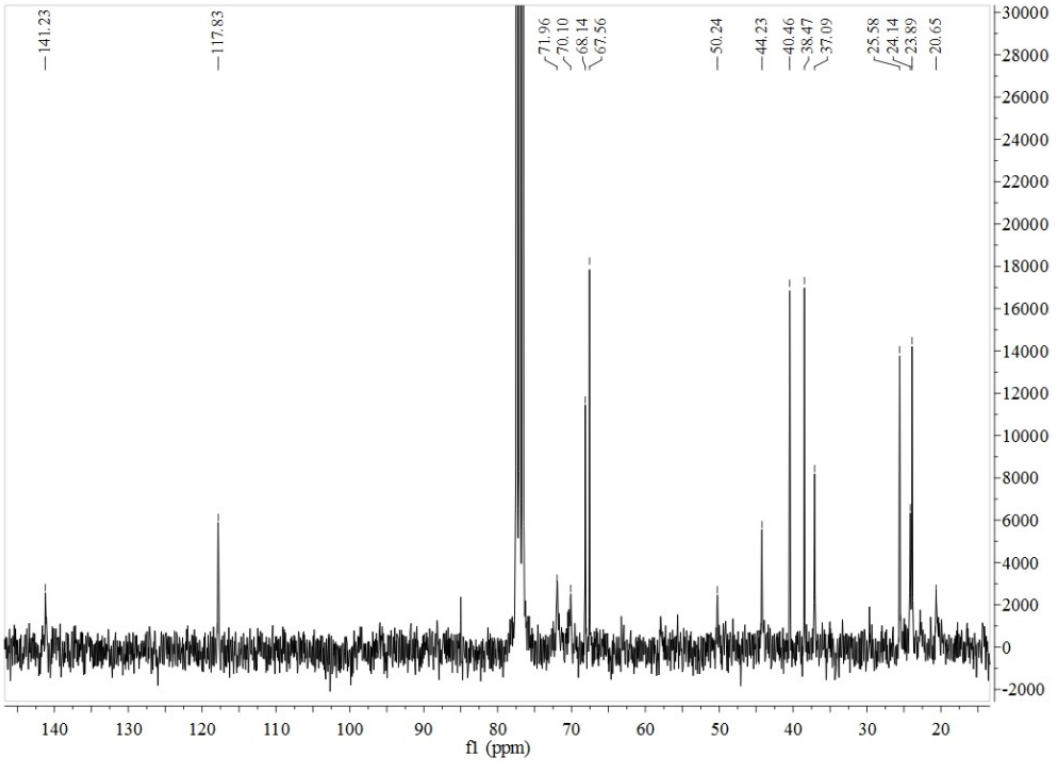

Figure 5. ^{13}C NMR spectra (75 MHz, $CDCl_3$) of (+)-obtusol.

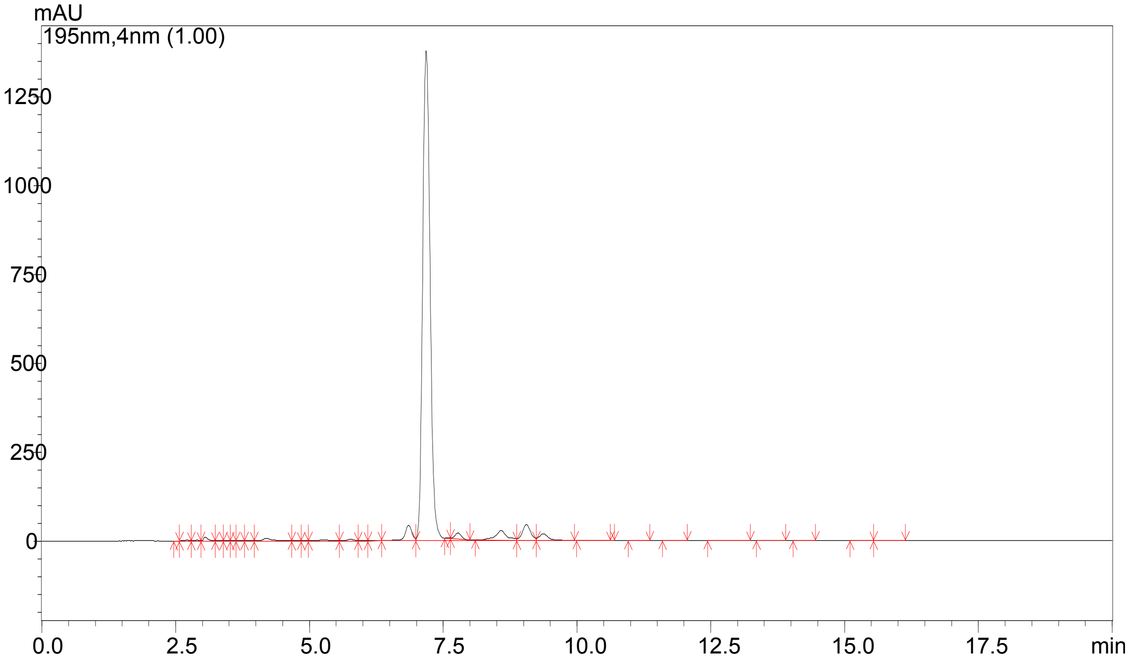

Figure 6. HPLC analysis of (+)-obtusol using a Shimadzu LC-20AT with the detector SPD-M20A and column Shim-pack VP-ODS (C18) 250 × 4.6 mm, 5 µM. Eluting system: acetonitrile 85%: de-ionized water (Milli-Q, Millipore) 15% , flow rate 1 mL/min.

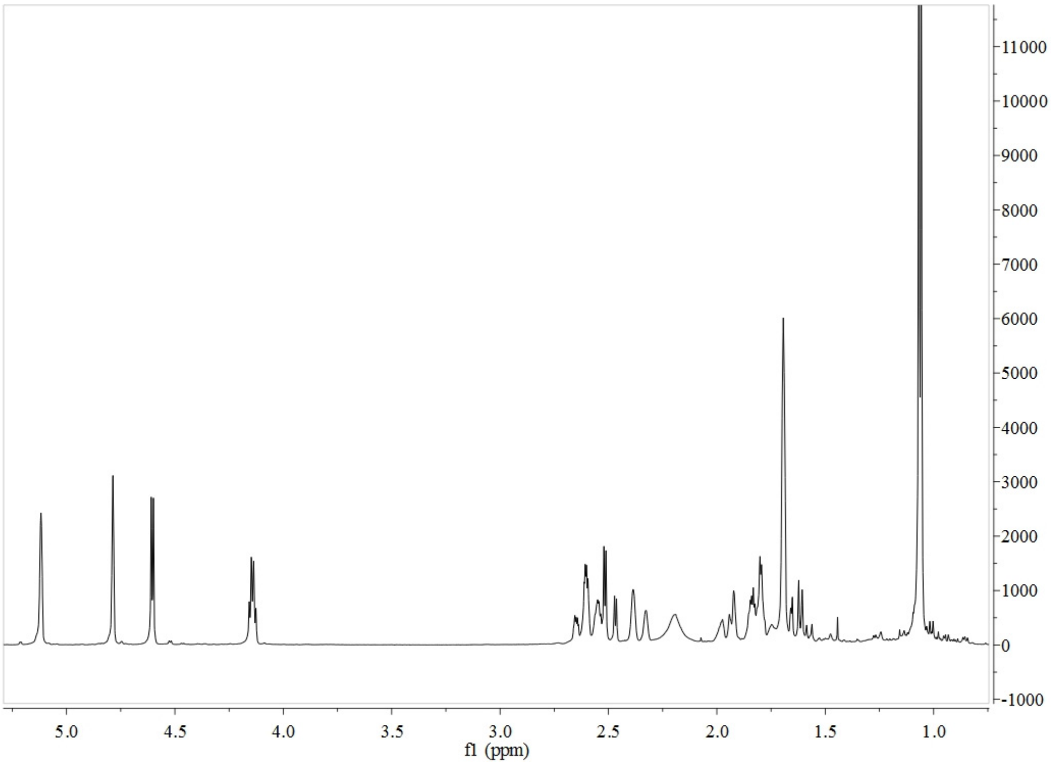

Figure 7. ^{1}H NMR spectra (300 MHz, $CDCl_3$) of (−)-elatol.

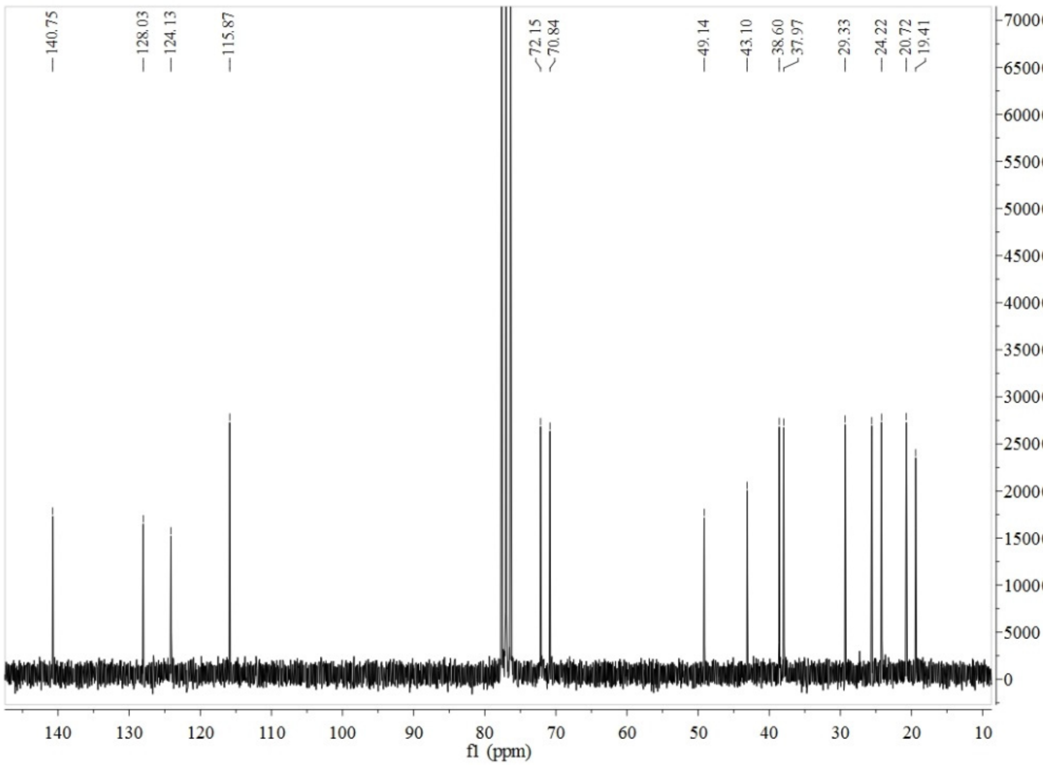

Figure 8. ^{13}C NMR spectra (75 MHz, $CDCl_3$) of (−)-elatol.
